# Genetic Variability, Trait Association, and Multi-Trait Selection of New Indeterminate Tomato Genotypes Under Protected Cultivation

**DOI:** 10.3390/plants15111760

**Published:** 2026-06-05

**Authors:** Ramya Shekhar, Awani Kumar Singh, Ramesh Kumar Yadav, Harshawardhan Choudhary, Ram Asrey, Gyan Prakash Mishra, Bhanushree Narayanswami, Paresh Chaukhande, K. G. Gainiamliu, Chaithra Mutthuraju, Rakesh Kumar, Saheb Pal, Chetna Shaktawat, Narendra Singh, Jogendra Singh

**Affiliations:** 1 ICAR-Indian Agricultural Research Institute, New Delhi 110012, India; ramyashekhar1997@gmail.com (R.S.); gyan.gene@gmail.com (G.P.M.); paresh.chaukhande@gmail.com (P.C.);; 2 ICAR-Research Complex for Eastern Region, FSRCHPR, Ranchi 834010, India; 3 ICAR-IIHR, Central Horticultural Experiment Station, Chettali 571248, India; 4 ICAR-Indian Agricultural Research Institute, Hazaribagh 825405, India; 5 Department of Horticulture, Swami Keshwanand Rajasthan Agricultural University, Bikaner 334006, India

**Keywords:** indeterminate tomato, fruit traits, heritability, pre-breeding, protected cultivation

## Abstract

Tomato is an important vegetable crop suited to both open-field and protected cultivation. Indeterminate genotypes with high yield potential and desirable quality traits are especially suited to off-season production under protected cultivation. The present study evaluated 57 indeterminate tomato genotypes over two consecutive years under protected conditions to assess genetic variability, genetic divergence, and trait associations across 16 important yield-attributing and quality traits. The analysis of variance depicted significant differences among genotypes for all traits under study. The traits, viz., fruit weight and number of fruits per cluster, exhibited high heritability and high genetic gain, suggesting the predominance of additive gene action and the possibility of direct selection. A significant, positive correlation between fruit weight and the number of plant clusters and yield was observed. Analysis of genetic divergence following Mahalanobis D^2^ statistics classified the genotypes into seven clusters. The number of flowers per cluster and fruit width were the top contributors to the total genetic divergence. Cluster VI outperformed for earliness and yield, Cluster V outperformed for nutritional quality, while Cluster VII was superior for fruit size. Principal Component Analysis revealed that the first five components cumulatively explained 83.3% of the total variation, with PC1 defined by fruit number trait and PC2 by yield and earliness traits. The Multi-Trait Genotype-Ideotype Distance Index (MGIDI) was used to select the best-performing genotypes, highlighting PIDGT-39, PIDGT-42, and PIDGT-29 as elite. Thus, the findings of the present study provide deeper insights into the genetic makeup of indeterminate tomato genotypes and potential parental accessions for tomato improvement, to enhance yield and quality under protected conditions.

## 1. Introduction

Tomato (*Solanum lycopersicum* L.) is one of the most widely cultivated solanaceous vegetable crops throughout the globe. It is native to Central and South America. In India, tomato is cultivated on approximately 0.854 million hectares, with an annual production of about 23.32 million tonnes during 2024–2025, although a major proportion of production still occurs under open-field conditions. It is rich in essential components, including vitamins A and C, minerals, sugars, organic acids, and lycopene [1,2]. Additionally, it contains compounds with antioxidant activity, including chlorogenic acid, plastoquinones, rutin, tocopherol, and xanthophylls. Thus, tomato is considered a “protective food” because it reduces the risk of certain cancers, helps maintain blood pressure, and regulates blood glucose levels [3].

In recent years, agricultural production systems have shifted towards protected cultivation, ensuring high-quality production and aiming to achieve food and nutritional security. Protected environments favorably modify microclimatic parameters, such as temperature, humidity, and light intensity, around plants to meet their requirements, thereby safeguarding them from various biotic and abiotic stresses [4,5]. This transition has necessitated identifying desirable indeterminate genotypes that better utilize vertical space for extended harvest periods and are compatible with high-density planting, training, and pruning systems [6].

Recent breeding efforts at the ICAR-Indian Agricultural Research Institute (ICAR-IARI), New Delhi, have led to the development of several advanced tomato inbred lines with improved fruit yield, quality, and adaptability to protected cultivation in India. These inbred lines were developed from genetically diverse tomato germplasm resources and represent valuable breeding materials for intensive production systems. However, their performance has not yet been systematically evaluated across seasons under naturally ventilated polyhouse conditions. Therefore, a comprehensive multi-season evaluation of these inbred lines is essential to assess their yield potential, fruit-quality attributes, stability, and overall suitability for commercial cultivation and future breeding programs in protected environments. Furthermore, the success of trait-specific breeding programs largely depends on the existence of sufficient genetic variability within the germplasm [4]. The range of this variability present in germplasm can be assessed using phenotypic and genotypic coefficients of variation. Traits with high heritability estimates are less influenced by environmental factors, making them reliable for selection in controlled environments [5]. However, high heritability alone is not sufficient for efficient selection in segregating generations; it must be accompanied by substantial genetic advance [5].

The increasing demand for premium-quality fruits with higher productivity, extended shelf life, improved nutritional quality, and adaptability to protected environments has intensified tomato breeding efforts globally [5,7]. Breeding objectives in tomato largely depend on the intended end use of the fruit, including fresh market consumption, salad purposes, processing, cherry tomato production, and long-distance transportation. Tomato fruit size is an important market-driven trait, with medium-sized fruits generally preferred for the fresh market, small-sized fruits favored for salad use, and larger fruits suitable for processing. However, in indeterminate tomato breeding under protected cultivation, breeding objectives extend beyond fruit size and include earliness, high yield potential, extended harvesting duration, fruit uniformity, attractive shape and color, firmness, shelf life, and superior nutritional quality, all of which determine market acceptance and profitability [6,7,8]. In addition, indeterminate genotypes should possess desirable plant architecture, sustained fruit set, and efficient assimilate partitioning to support continuous production over a longer cropping period. Therefore, understanding the nature and magnitude of associations among yield, earliness, and related component traits is essential for designing effective breeding strategies to simultaneously improve productivity, fruit quality, and market-oriented traits in greenhouse-grown indeterminate tomato genotypes.

In this context, the present investigation was undertaken to evaluate 57 new indeterminate tomato genotypes under protected cultivation. The objectives were to assess genetic variability, determine associations between yield and its component traits, and identify superior genotypes using the Multi-Trait Genotype-Ideotype Distance Index (MGIDI) to enhance productivity under protected cultivation systems.

## 2. Materials and Methods

### 2.1. Plant Materials

The experimental material comprised 57 indeterminate tomato inbred lines maintained at the Center for Protected Cultivation and Technology (CPCT), ICAR-Indian Agricultural Research Institute (ICAR-IARI), New Delhi, India. These inbred lines were developed through successive selfing and selection from genetically diverse indigenous tomato collections maintained at ICAR-IARI. The source germplasm represented a broad range of variability for plant growth, fruit morphology, fruit quality traits, and adaptation to protected cultivation conditions. During the inbreeding process, selections were made over successive generations based on plant vigor, earliness, fruit set, yield potential, fruit size and shape, marketable fruit quality, and overall agronomic performance under protected cultivation. The resulting advanced inbred lines were subsequently maintained through self-pollination and used for the present evaluation.

The present experiment was conducted at the CPCT, ICAR-IARI, New Delhi, over two years (November 2022 to May 2023 and November 2023 to May 2024) in a naturally ventilated polyhouse under the following conditions. The experimental field is located at 28.63° N, 77.16° E, and approximately 228.6 m above mean sea level. The genotypes were evaluated in a randomized block design with three replications. Healthy, uniform seedlings were raised in trays using a sterilized growing medium and transplanted into the polyhouse. Inside the polyhouse, the average temperature ranged from 18 to 32 °C with relative humidity between 60–85% during the crop growth period under natural photoperiod conditions. Standard greenhouse production practices, including staking, pruning, fertigation, irrigation scheduling, plant protection measures, and canopy management, were uniformly followed across all treatments to ensure optimal crop growth and minimize environmental variation among experimental units, in accordance with recommended protected cultivation protocols. Each experimental unit consisted of 15 plants per genotype per replication. Observations were recorded on five randomly selected healthy, representative plants, for 16 agro-morphological and quality characters in accordance with the DUS guidelines for tomato.

### 2.2. Plant Growth and Yield Parameters

Plant growth parameters, viz., plant height (cm), days to 50% flowering, number of flowers per cluster, number of clusters per plant, number of fruits per cluster, and number of fruits per plant were recorded as per standard methodology [9]. Plant height (cm) was measured from the base of the plant at ground level to the apical growing point at the end of the flowering period using a measuring scale. Days to 50% flowering were recorded as the number of days from transplanting to the stage when 50% of plants in each genotype produced at least one open flower. The number of flowers per cluster was determined by counting the total flowers produced in randomly selected clusters and calculating the average. The total number of clusters per plant was counted at the end of the cropping period. The number of fruits per cluster was recorded by counting fruits set in selected clusters at harvest and expressed as an average. The total number of fruits per plant was obtained by counting all harvested fruits from each selected plant during the cropping period, and the mean value was calculated. Fruit yield per plant (kg) was calculated by summing the total fruit weight harvested from each plant across all pickings during the cropping period. The cumulative fruit weight per experimental unit was divided by the total number of plants to obtain the average fruit yield per plant, which was expressed in kilograms.

### 2.3. Fruit Physical Parameters

Fruit physical parameters, including fruit length (cm), fruit width (cm), pericarp thickness (cm), fruit weight (g), locules per fruit, and yield per plant (kg), were recorded using standard procedures [9]. Observations were made on ten randomly selected, uniform, and marketable fruits harvested at the red-ripe stage from the fourth harvest of each genotype, and mean values were used for statistical analysis. Fruit weight (g) was determined individually using a calibrated digital electronic balance (CTG 3101 Precision Balance, Citizen Scale Pvt. Ltd., Mumbai, India; sensitivity: 0.1 g). Fruit length (cm) was measured as the longitudinal distance from the pedicel attachment point to the blossom end of the fruit using a digital vernier caliper (Model 500-196, Mitutoyo South Asia Pvt. Ltd., New Delhi, India; Sensitivity 0.01 mm), while fruit width (cm) was measured as the equatorial diameter at the widest central portion of the fruit.

For the determination of locules per fruit and pericarp thickness (cm), fruits were cut transversely at the equatorial region using a sharp stainless-steel blade. The number of locules present in each fruit was counted manually. Pericarp thickness was measured using a digital vernier caliper at three equidistant points along the cut surface of each fruit.

### 2.4. Fruit Biochemical Parameters

Total soluble solids (TSS) content of tomato fruits was determined using freshly extracted clear fruit juice with the help of a calibrated handheld digital refractometer (BRIX 0–32%, Erma Inc., Yoshikawa, Tokyo) at room temperature (25 ± 1.0 °C). The refractometer was calibrated with distilled water before each set of observations. The TSS content was expressed as degrees Brix, representing the percentage of soluble solids present in the fruit juice, and the mean of replicate observations was recorded. Titratable acidity was determined by titrimetric analysis following the standard procedure described by Ranganna [10]. Fresh tomato juice was extracted from ripe fruits by homogenization and filtration through muslin cloth. 10 mL of juice was diluted with distilled water and titrated against a 0.1 N sodium hydroxide (NaOH) solution using phenolphthalein as an indicator until a persistent pale pink endpoint color was observed. Titratable acidity was calculated as citric acid equivalent and expressed as percentage.

Ascorbic acid content was estimated by the dye titration method [10]. 10 g of fresh fruit pulp was homogenized with 3% metaphosphoric acid and filtered to obtain a clear extract. An aliquot of the extract was titrated against standardized 2,6-dichlorophenol indophenol dye until a light pink colour persisted for approximately 15 s.

Lycopene content was determined following the spectrophotometric method [11]. A 1 g sample of fresh ripe tomato pulp was homogenized with 8 mL of hexane:acetone:ethanol (2:1:1, *v*/*v*/*v*) using a mortar and pestle until complete pigment extraction. The homogenate was transferred to a test tube, and 1 mL of distilled water was added to facilitate phase separation. The mixture was shaken thoroughly and allowed to stand for 10 min at room temperature until two distinct layers were formed. The upper hexane layer, containing lycopene and other carotenoids, was carefully collected, and its absorbance was measured at 503 nm using a UV–Visible spectrophotometer against hexane as the blank. Lycopene content was calculated using the lycopene extinction coefficient in hexane and expressed as mg per 100 g of fresh weight of tomato pulp.

### 2.5. Statistical Analysis

The morphological parameters recorded over both years were pooled to minimize seasonal effects and were subjected to statistical analysis. Analysis of Variance (ANOVA) was performed to detect genotypic differences. Genetic parameters, including genotypic and phenotypic coefficients of variation (GCV and PCV, respectively), broad-sense heritability (h^2^), and genetic advance over percent mean (GAM, or genetic gain), were estimated using standard biometrical procedures [12]. The correlation was performed as described by Al-Jibouri [13]. The genotypic correlation coefficients were further bifurcated into direct and indirect effects through path coefficient analysis. Genetic divergence among genotypes was assessed using Mahalanobis D^2^ statistics, and clustering was performed using Tocher’s method. Principal Component Analysis (PCA) and the Multi-Trait Genotype-Ideotype Distance Index (MGIDI) were performed using the “metan” package in R version 4.5.1 (R Foundation for Statistical Computing, Vienna, Austria) [14]. All other statistical analyses were also performed using R software version 4.5.1.

For MGIDI analysis (“metan” package of the R software), traits related to productivity, fruit quality, and nutritional attributes, including yield per plant, fruit weight, fruit dimensions, number of fruits per plant, number of clusters per plant, total soluble solids, ascorbic acid, and lycopene content, were considered as traits to be increased (higher values preferred). Days to 50% flowering was considered a trait to be decreased (lower values preferred) to favor. All traits were rescaled and assigned equal weight in the MGIDI analysis. All traits were assigned equal importance (equal weight) during MGIDI computation, and trait values were rescaled according to the predefined selection direction before factor analysis [9,15].

## 3. Results

### 3.1. Genetic Variability Studies

The pooled analysis of variance across two consecutive years revealed significant differences among the evaluated tomato genotypes for all studied traits under naturally ventilated polyhouse conditions (Table 1). Significant genotype × environment interactions were also observed for several traits. High variance estimates were recorded for the number of fruits per plant (1736.608), plant height (49.086 cm), and fruit weight (25.164 g).

Substantial variability was observed among the genotypes for growth, yield, and fruit morphological traits. The number of fruits per plant ranged from 19.48 (PIDGT-12) to 331.75 (PIDGT-54). Fruit length varied from 2.25 cm (PIDGT-63) to 6.32 cm (PIDGT-2), while fruit width ranged from 2.06 cm (PIDGT-53) to 7.92 cm (PIDGT-38). Pericarp thickness ranged from 0.14 cm (PIDGT-51) to 1.12 cm (PIDGT-2). Average fruit weight varied from 7.92 g (PIDGT-54) to 164.92 g (PIDGT-38) (Table S1).

Significant variation was also recorded for the number of locules per fruit. Yield per plant ranged from 0.65 kg to 10.28 kg among the newly developed indeterminate tomato lines evaluated. Considerable variation was also observed for fruit biochemical and quality traits (Table S2). Total soluble solids (TSS) ranged from 4.56 °Brix (PIDGT-14) to 8.37 °Brix (PIDGT-60). Lycopene content varied from 7.18 mg/100 g (PIDGT-39) to 14.98 mg/100 g (PIDGT-54). Titratable acidity ranged from 0.26% (PIDGT-11) to 0.77% (PIDGT-51 and PIDGT-57). Ascorbic acid content ranged from 13.32 mg/100 g (PIDGT-39) to 41.59 mg/100 g (PIDGT-61).

The estimates of genotypic coefficient of variation (GCV) and phenotypic coefficient of variation (PCV) are presented in Table 2. For all studied traits, PCV values were higher than the corresponding GCV values. High GCV and PCV estimates were observed for most yield- and fruit-related traits. In contrast, comparatively lower estimates were observed for plant height, days to 50% flowering, TSS, and lycopene content. Moderate GCV and PCV values were recorded for lycopene content (18.65 and 18.94, respectively), TSS (18.57 and 19.00, respectively), and days to 50% flowering (10.19 and 10.34, respectively). Plant height recorded the lowest GCV and PCV values (7.67 and 7.85, respectively). High heritability estimates (>60%) were observed for all evaluated traits. The highest heritability values were recorded for fruit width (99.58%), fruit weight (99.24%), fruit length (98.95%), number of locules per fruit (98.94%), pericarp thickness (98.88%), ascorbic acid content (98.64%), lycopene content (96.99%), days to 50% flowering (96.97%), number of clusters per plant (96.73%), titratable acidity (96.42%), plant height (95.66%), TSS (95.58%), number of fruits per plant (94.34%), number of flowers per cluster (91.67%), number of fruits per cluster (89.24%), and yield per plant (88.42%).

Furthermore, genetic advance varied considerably among the studied traits, ranging from 0.23 for titratable acidity to 138.96 for the number of fruits per plant. High genetic advance as percent of mean (GAM) was recorded for number of fruits per plant (180.01), yield per plant (142.89), number of fruits per cluster (118.91), fruit weight (102.27), number of flowers per cluster (99.40), ascorbic acid content (70.89), pericarp thickness (70.54), number of locules per fruit (60.81), fruit width (56.49), titratable acidity (51.91), number of clusters per plant (48.22), fruit length (41.14), lycopene content (37.84), TSS (37.40), and days to 50% flowering (20.66). Plant height recorded comparatively lower genetic advance.

### 3.2. Correlation Studies

Yield is a polygenic trait, regulated by complex interactions among physiological and morphological traits. Consequently, direct selection for yield is often rendered inefficient due to its complex relation with other plant morphological and yield-contributing traits. Thus, breeders rely on indirect selection via component traits with high heritability and strong association with yield. In the present study, genotypic correlation coefficients were estimated to determine the nature and magnitude of associations among the 16 growth and fruit quality parameters and fruit yield (Table 3). The correlation analysis revealed strong associations among several characters, indicating interdependent relationships between yield-contributing and biochemical traits in the evaluated new tomato lines.

Yield per plant exhibited significant positive correlations with fruit weight (0.617), number of clusters per plant (0.340), fruit length (0.231), and pericarp thickness (0.234). In contrast, yield per plant showed a significant negative correlation with days to 50% flowering (−0.229). Fruit weight was positively associated with fruit length and fruit width, whereas the number of clusters per plant showed a positive association with yield and fruit number-related traits. Plant height exhibited significant negative correlations with days to 50% flowering, fruit width, and number of locules per fruit, while positive correlations were observed with total soluble solids (TSS) and titratable acidity. Days to 50% flowering showed positive associations with fruit length and fruit weight, but a negative association with the number of clusters per plant. The number of fruits per plant and the number of fruits per cluster exhibited strong positive correlations with each other and with biochemical quality traits, including TSS, titratable acidity, and ascorbic acid content. However, these traits showed negative correlations with fruit weight, length, width, pericarp thickness, and the number of locules per fruit. Biochemical traits also exhibited notable associations among themselves. TSS and titratable acidity were positively correlated with each other but negatively associated with fruit weight and yield-related traits. Lycopene and ascorbic acid content showed variable associations with fruit morphological and yield parameters across the evaluated genotypes.

### 3.3. Path Coefficient Analysis

The correlation coefficients quantify the degree of association between two variables. However, it provides limited insights into the cause-and-effect relationships. A trait may show a strong positive correlation with yield simply because it is associated with another trait that directly influences yield. Therefore, to understand such complex interrelation, path coefficient analysis was performed to partition the observed genotypic correlations into their direct and indirect effects on tomato yield.

The results of the path analysis at the genotypic level are presented in Table 4. Among the evaluated traits, the number of clusters per plant exhibited the highest positive direct effect on yield per plant (0.8745), followed by fruit weight (0.8627). Fruit length and pericarp thickness also showed positive direct contributions toward yield, although their effects were comparatively smaller. The positive direct effects of these traits were consistent with their significant positive correlations with yield. The number of fruits per plant exerted a strong negative direct effect on yield (−0.891), despite showing positive indirect effects through the number of fruits per cluster and other related traits. Similarly, plant height and days to 50% flowering exhibited negative direct effects on yield per plant. Among fruit-quality traits, total soluble solids (TSS) and titratable acidity showed negative direct effects on yield, whereas ascorbic acid content exhibited a marginally positive direct effect. Several traits also contributed indirectly to yield through their associations with fruit weight and cluster-related characters. The residual effect from the analysis was low (0.254), indicating that the traits included in the present study collectively explained a large proportion of the total variability in fruit yield per plant. The path analysis identified the number of clusters per plant and fruit weight as the major direct contributors to yield, highlighting their importance as potential selection criteria for improving productivity in indeterminate tomato genotypes under protected cultivation conditions.

### 3.4. Principal Component Analysis (PCA)

Principal Component Analysis (PCA) was performed to assess the multivariate relationships among the evaluated tomato genotypes and to reduce the dataset’s dimensionality. The first five principal components exhibited eigenvalues greater than 0.8 and collectively accounted for 83.3% of the total variation present in the evaluated indeterminate tomato lines (Table 5).

Among these, the first principal component (PC1) explained 50.5% of the total variance, whereas the second principal component (PC2) contributed an additional 13.1% of the total variation. The scree plot showed a sharp decline in explained variance after the first principal component, indicating that most of the variability was captured by the first few dimensions (Figure S1). Reducing complex data into interpretable components enables breeders to visualize the genotype structure effectively. Trait loadings revealed that PC1 was mainly associated with the number of fruits per plant (1.35), the number of fruits per cluster (1.32), the number of flowers per cluster (1.29), and total soluble solids (TSS). PC2 was primarily influenced by yield per plant (15.10), days to 50% flowering (11.86), and plant height (6.90). The fourth and fifth principal components accounted for comparatively small proportions of the total variation and were primarily associated with biochemical traits, including lycopene, ascorbic acid, TSS, and titratable acidity (Table 6).

**Figure 1:**
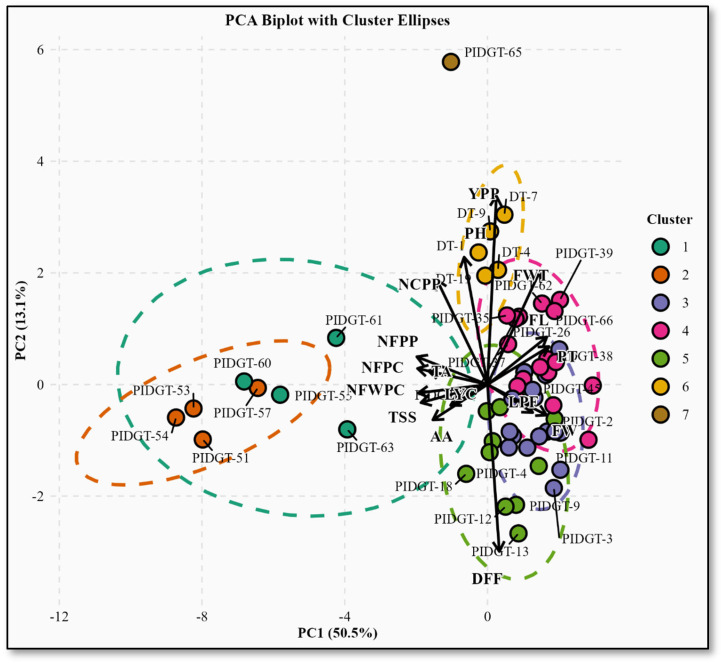
PCA biplot showing the distribution of 57 genotypes evaluated for two consecutive years under protected conditions for different agro-morphological and fruit-quality traits with cluster ellipses.

The PCA biplot showed a distinct spatial distribution of the evaluated genotypes based on fruiting behaviour, yield performance, and fruit-quality traits (Figure 1). The horizontal axis primarily separated prolific small-fruited genotypes from large-fruited accessions, whereas the vertical axis differentiated high-yielding genotypes from relatively low-yielding types. The monotypic genotype PIDGT-65 occupied a distinct position in the biplot due to its extreme fruit weight and yield characteristics. The distribution pattern of fruit-quality traits and yield-related parameters in the biplot indicated differential associations among productivity and biochemical attributes within the evaluated lines.

### 3.5. Genetic Diversity in Tomato Genotypes

**Figure 2:**
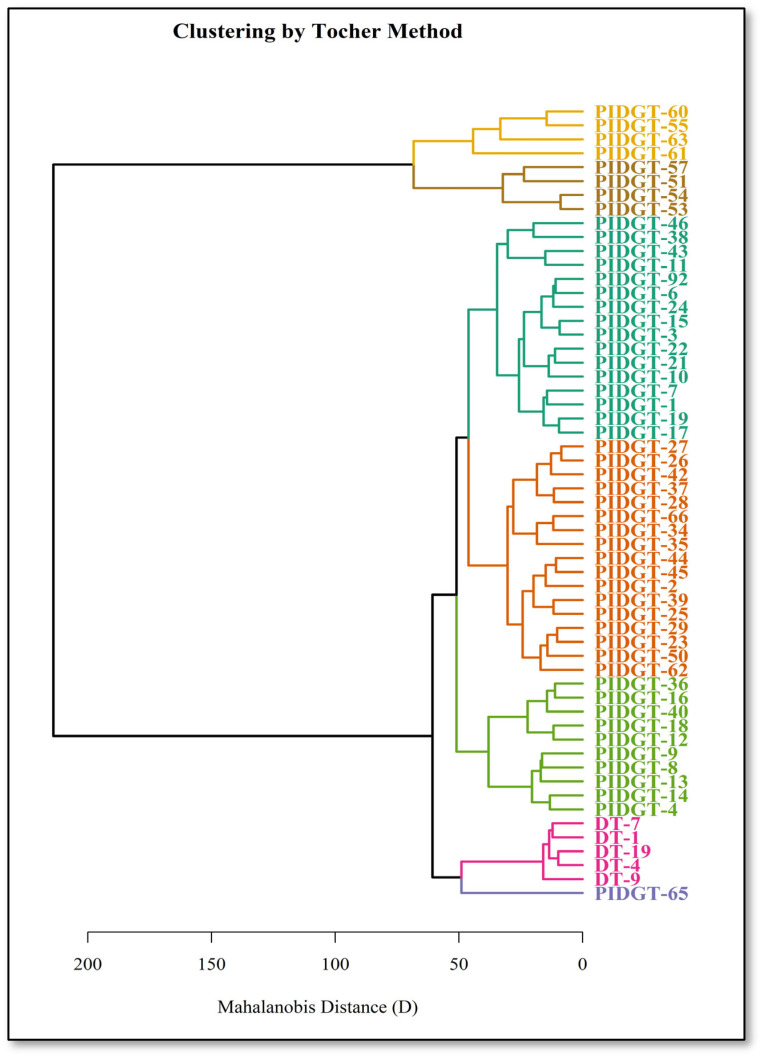
Dendrogram depicting the clustering pattern of 57 tomato genotypes formed following Tocher’s method using data of agro-morphological, yield and fruit-quality traits.

The assessment of genetic divergence is a mainstay of modern plant breeding, providing a quantitative basis for selecting diverse parents to maximize hybrid vigour in breeding programs. In this study, genetic divergence among the evaluated tomato genotypes was assessed using Mahalanobis D^2^ statistics, and the genotypes were grouped into seven distinct clusters following Tocher’s method (Figure S2). The clustering pattern in the dendrogram (Figure 2) shows an asymmetrical distribution of genotypes, indicating non-random genetic diversity. Cluster IV was the largest cluster, comprising 17 genotypes, followed by Cluster III, containing 16 genotypes. Cluster VI consisted of five genotypes, predominantly belonging to the DT series (DT-1, DT-19, DT-4, DT-7, and DT-9). Cluster VII was monotypic, containing only PIDGT-65, indicating substantial divergence of this genotype from the other genotypes.

**Figure 3:**
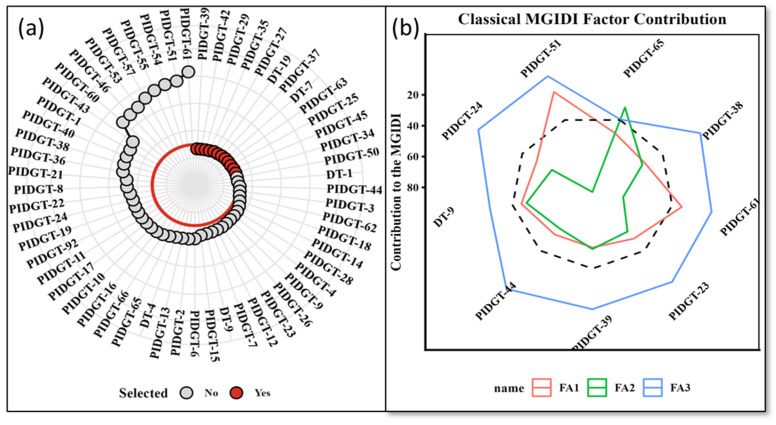
MGIDI-based selection of superior tomato genotypes under protected cultivation: (**a**) circular genotype ranking relative to the ideotype and (**b**) factor contribution plot showing the strengths and weaknesses of selected genotypes.

The average intra- and inter-cluster distances are presented in Figure 3. Inter-cluster distances were consistently higher than intra-cluster distances, indicating the presence of substantial genetic divergence among clusters. The maximum inter-cluster distance (D^2^ = 7394.68) was observed between Cluster II and Cluster VII, followed by Cluster II and Cluster III (D^2^ = 5977.06). Considerable divergence was also observed between Clusters V and VI.

Cluster mean analysis demonstrated substantial variation among clusters for yield and fruit-quality traits (Table 7). Cluster VI recorded the highest mean yield per plant (6.47 kg) along with the earliest flowering (34.92 days). Cluster V exhibited higher mean values for total soluble solids, lycopene, and ascorbic acid content. The monotypic Cluster VII containing PIDGT-65 recorded exceptionally high fruit weight (159.42 g) and fruit number. The relative contribution of individual traits toward total genetic divergence is presented in Table 8. The number of flowers per cluster contributed the highest proportion (23.43%) toward total divergence, followed by fruit width (15.29%). Several fruit morphological, reproductive, and quality traits also contributed substantially to overall genetic differentiation among the evaluated genotypes.

### 3.6. MGIDI-Based Selection

The Multi-Trait Genotype-Ideotype Distance Index (MGIDI) was used to identify superior tomato genotypes by simultaneously evaluating 17 agro-morphological, yield, and fruit-quality traits. The MGIDI ranked genotypes by their Euclidean distance from the defined ideotype, with lower MGIDI values indicating greater similarity to the ideal genotype. The genotypic rankings are presented in Figure 3, and the scores of the top-performing genotypes are presented in Table 9.

Among the evaluated genotypes, PIDGT-39 recorded the lowest MGIDI score (13.32), followed closely by PIDGT-42 (13.33) and PIDGT-29 (13.46). Other superior genotypes identified through the MGIDI included PIDGT-35, PIDGT-27, and DT-19. These genotypes exhibited favourable combinations of yield, fruit morphology, and quality traits under naturally ventilated polyhouse conditions. The efficiency of MGIDI-based selection was assessed by comparing the mean performance of the selected genotypes with the overall means across all studied traits (Table S3). The selected genotypes exhibited a 16.28% increase in average fruit weight and a 14.00% increase in yield per plant over the overall mean. Plant height also increased by 5.55% in the selected group. In contrast, days to 50% flowering showed a reduction of 2.60%, indicating comparatively earlier flowering among the selected genotypes. Furthermore, the selected genotypes exhibited lower mean values for ascorbic acid (−33.95%) and lycopene content (−11.33%) compared with the overall mean. Total soluble solids, titratable acidity, and other fruit-quality traits showed variable responses among the selected genotypes. The strengths and weaknesses of the selected genotypes were further evaluated using the factor contribution plot (Figure 3). The radar plot revealed differences among genotypes for specific trait groups. Genotypes such as PIDGT-39 exhibited balanced performance across multiple trait categories, whereas certain accessions displayed comparatively weaker performance for fruit-quality traits.

## 4. Discussion

### 4.1. Genetic Variability and Selection Potential

The significant variation observed among the evaluated tomato genotypes across all studied traits under naturally ventilated polyhouse conditions confirms the presence of substantial exploitable genetic diversity. Similar extensive variability for yield and fruit-quality traits in tomato germplasm has also been reported previously [16,17,18,19,20]. Marked variation was observed for major yield-contributing traits, including the number of fruits per plant, fruit weight, fruit dimensions, and yield per plant. The exceptionally wide range in fruit number and fruit weight reflects the coexistence of small-fruited and large-fruited genotypes among the evaluated genotypes. Such broad variability is fundamental for effective selection and genetic improvement in tomato breeding programs for protected cultivation systems [7,21,22,23].

Considerable diversity was also observed in fruit quality and biochemical parameters, including total soluble solids (TSS), lycopene, titratable acidity, and ascorbic acid content, which are consistent with previous findings in tomato [19,23,24]. The observed variation in TSS and acidity is highly relevant for processing efficiency and consumer acceptability, whereas variation in lycopene and ascorbic acid content reflects substantial differences in nutritional and antioxidant potential among genotypes [25,26]. The observed variability provides opportunities for simultaneous improvement of processing quality and nutritional attributes through targeted breeding. The wide variation in these biochemical traits further indicates diverse metabolic backgrounds governing sugar accumulation, organic acid biosynthesis, and antioxidant metabolism in tomato fruits [3,26].

The consistently higher phenotypic coefficient of variation (PCV) compared with the corresponding genotypic coefficient of variation (GCV) across traits suggests the influence of environmental factors on trait expression; however, the relatively narrow differences between PCV and GCV for most characters indicate substantial genetic influence on trait expression with comparatively lower environmental interference under the evaluated conditions. High estimates of both PCV and GCV across most traits indicate substantial inherent variability and a broad scope for phenotypic selection. Moderate GCV and PCV estimates for TSS, lycopene, and days to 50% flowering suggest the involvement of both genetic and environmental factors in regulating these traits. Previously, Cuartero and Cubero [27] also reported that earliness was the most environmentally dependent characteristic among the other fruit and yield traits in tomato. Such moderate variability suggests that improvement through direct selection may remain effective when combined with hybridization and subsequent selection in segregating generations [27]. In contrast, the relatively low variability observed in plant height might be attributed to the exclusive inclusion of indeterminate genotypes with comparatively uniform growth habits under protected growing conditions [20].

The high heritability estimates recorded for all studied traits indicate predominance of genetic effects over environmental influences in trait expression. Extremely high heritability for fruit width, fruit weight, fruit length, number of locules per fruit, pericarp thickness, ascorbic acid content, lycopene content, and yield-related traits suggests that these characters are largely governed by transmissible genetic factors and can therefore be effectively improved through phenotypic selection. Furthermore, a high magnitude of genetic advance as percent of mean (GAM) for number of fruits per plant, yield per plant, fruit weight, number of fruits per cluster, and ascorbic acid content further supports the presence of substantial additive genetic variance for these traits. Similar findings have been reported in previous studies for fruit yield and quality traits [28,29]. High heritability, coupled with substantial genetic advance for most traits, suggests the likely involvement of additive genetic effects, indicating that phenotypic selection may be effective for improving these traits [21,30,31]. The present findings showed substantial genetic variability for yield, fruit morphology, and biochemical quality traits within the evaluated genotypes, highlighting their potential utility for varietal development and parental selection in future tomato breeding programs for protected cultivation systems.

### 4.2. Trait Association and Yield Determination

Tomato fruit yield is a highly complex quantitative trait governed by interactions among multiple morphological, physiological, and biochemical components [8,27]. Therefore, understanding the interrelationships among yield-contributing traits is essential for designing effective selection strategies in breeding programmes [20,23,30].

The correlation analysis revealed strong positive associations of yield per plant with fruit weight, number of clusters per plant, fruit length, and pericarp thickness, indicating that these traits are major contributors to yield improvement in indeterminate tomato lines. These results are consistent with previous studies in tomato [19,31,32]. The significant negative association between yield and days to 50% flowering indicates that early flowering genotypes tend to exhibit superior productivity under protected cultivation. Earliness likely enhances cumulative harvest duration and improves resource-use efficiency by allowing earlier transition from vegetative to reproductive growth [30]. Plant height exhibited negative correlations with days to 50% flowering, fruit width, and number of locules per fruit, while maintaining positive associations with TSS and titratable acidity. Similar associations involving TSS, titratable acidity, and flowering behaviour have also been documented in previous studies [20,23]. These relationships suggest that taller indeterminate genotypes with efficient canopy architecture may enhance light interception and photosynthetic efficiency, thereby improving assimilate distribution and fruit biochemical quality [7]. Furthermore, days to 50% flowering showed positive associations with fruit size traits but a negative association with the number of clusters per plant, indicating a potential trade-off between earliness and fruit enlargement. The strong positive correlations observed among the number of fruits per plant, the number of fruits per cluster, TSS, titratable acidity, and ascorbic acid indicate close physiological and metabolic relationships among fruiting intensity and biochemical quality traits. However, these traits exhibited strong negative associations with fruit weight, fruit dimensions, pericarp thickness, and the number of locules per fruit, suggesting a possible trade-off between fruit number and individual fruit size within the evaluated lines [33]. Further, the positive association between TSS and titratable acidity, together with their negative relationship with fruit weight and yield, indicates the inherent difficulty of simultaneously improving these traits through conventional selection. Therefore, the simultaneous improvement of yield and fruit-quality traits may require advanced breeding approaches that exploit heterosis, use genomic-assisted selection, and employ strategic parental combinations [7].

Path coefficient analysis provided deeper insights into the direct and indirect contributions of component traits toward fruit yield. Among all traits studied, the number of clusters per plant exerted the highest positive direct effect on yield. Fruit weight also showed a strong positive direct effect, reaffirming its importance as a reliable selection criterion for yield improvement [16,31]. Although some negative indirect effects reduced the overall magnitude of the correlation between fruit weight and yield, its direct contribution remained consistently high. Notably, the number of fruits per plant had a strong negative direct effect on yield, despite positive indirect effects through cluster-related traits. This suggests that excessive fruit load results in smaller, less marketable fruits, thereby reducing overall productivity. Plant height and days to 50% flowering also exerted negative direct effects on yield, indicating that excessive vegetative growth and delayed reproductive transition may adversely affect assimilate allocation towards fruit development [7]. Among the quality traits, TSS and titratable acidity had negative direct effects on yield, whereas ascorbic acid showed a marginal positive direct effect, suggesting the possibility of simultaneous improvement in vitamin C content and productivity through careful selection. The relatively low residual effect obtained in the path analysis indicates that the evaluated traits collectively explained a major proportion of the total variability in fruit yield [34].

Overall, the combined correlation and path coefficient analyses identified fruit weight and number of clusters per plant as the most important selection criteria for improving yield in indeterminate tomato genotypes under protected cultivation conditions.

### 4.3. Multivariate Analysis and Genetic Divergence Among Tomato Genotypes

Multivariate analyses provide an effective framework for dissecting complex genetic relationships among genotype accessions and identifying key traits contributing to variability [17]. In the present study, Principal Component Analysis (PCA) efficiently reduced the dimensionality of the dataset and revealed substantial diversity among the evaluated tomato genotypes. Previously, PCA has been successfully employed to study key traits governing variation in different traits in tomato [35,36,37]. In our study, the first five principal components collectively explained 83.3% of the total variation, indicating that the selected agro-morphological and biochemical traits adequately captured the underlying genetic variability within the genotype panel. The high proportion of variance explained by the first two principal components further demonstrates the effectiveness of PCA in summarizing complex trait interactions into a smaller number of biologically meaningful dimensions.

The first principal component (PC1), which accounted for the largest proportion of the variability, was predominantly associated with fruit-number-related traits, including the number of fruits per plant, the number of fruits per cluster, the number of flowers per cluster, and TSS. This component largely differentiated prolific small-fruited genotypes from large-fruited accessions. In contrast, PC2 was mainly influenced by yield per plant, days to 50% flowering, and plant height, indicating that productivity and earliness were major contributors to the secondary axis of variation. The comparatively lower yet biologically important contributions of PC4 and PC5 were primarily associated with nutritional and biochemical traits, including lycopene, ascorbic acid, TSS, and titratable acidity. These findings suggest that fruit nutritional quality represents an independent dimension of variability within the evaluated genotypes. Similar observations were reported by Khor [25], who identified phytochemical parameters as major contributors to variability among tomato cultivars and emphasized their importance in identifying nutritionally superior genotypes. The PCA biplot exhibited the genetic relationships among the accessions by showing distinct spatial separation of genotype groups along the principal components. The horizontal axis primarily distinguished small-fruited, prolific genotypes from large-fruited types, whereas the vertical axis separated high-yielding genotypes from relatively low-yielding accessions. The distinct positioning of PIDGT-65 in a separate region of the biplot highlighted its unique genetic architecture, characterized by exceptionally high fruit weight and yield potential. Similar utility of PCA and biplot analyses for identifying genetically distinct tomato accessions has been demonstrated by Sivakumara et al. [38]. An important observation from the biplot analysis was the relatively distinct positioning of fruit-quality traits from yield-related traits, suggesting a weak relationship between productivity and biochemical quality. Similar trade-offs between yield and nutritional quality have previously been reported in tomato [26,39,40].

The Mahalanobis D^2^ analysis further confirmed substantial genetic divergence among the evaluated genotypes and grouped them into seven distinct clusters. The asymmetrical distribution of genotypes across clusters indicates non-random genetic diversity within the evaluated tomato genotypes, which is advantageous for parental selection in breeding programmes. Similar clustering patterns in tomato germplasm have been reported by Rasheed et al. [21]. The occurrence of a monotypic cluster containing PIDGT-65 further emphasizes the existence of highly distinct genotypes with unique combinations of economically important traits.

The higher magnitude of inter-cluster distances compared with intra-cluster distances confirmed the robustness of clustering and the existence of substantial divergence among clusters. The maximum inter-cluster distance observed between Cluster II and Cluster VII indicates highly diverse genetic backgrounds and suggests the possibility of broader recombination and potentially higher heterotic responses through hybridization between these groups. Similarly, the large divergence between Cluster VI and Cluster V suggests the feasibility of combining superior yield potential with enhanced fruit-quality attributes through strategic crossing programmes. Cluster mean analysis further highlighted the breeding significance of individual groups. Cluster VI emerged as an important source of high yield and earliness, whereas Cluster V possessed superior biochemical quality traits, including TSS, lycopene, and ascorbic acid content. The monotypic Cluster VII represented a valuable source of high fruit weight and sink capacity. The contrasting cluster characteristics offer useful opportunities to select complementary parents to combine productivity and nutritional quality in future breeding programs [8,21]. The contribution analysis identified the number of flowers per cluster and fruit width as major contributors to total genetic divergence, indicating the importance of reproductive efficiency and fruit morphology in differentiating tomato genotypes under protected cultivation conditions.

### 4.4. Multi-Trait Selection Efficiency Using MGIDI

Simultaneous improvement of yield, fruit quality, and agronomic performance remains one of the major challenges in tomato breeding because most economically important traits are quantitatively inherited and often exhibit complex interrelationships [16,17,32,41]. In the present study, the Multi-Trait Genotype-Ideotype Distance Index (MGIDI) effectively identified superior genotypes by integrating multiple agro-morphological and biochemical traits into a single selection criterion. The index ranked genotypes based on their proximity to a predefined ideotype, thereby enabling balanced multi-trait selection under protected cultivation conditions.

Among the evaluated genotypes, PIDGT-39 recorded the lowest MGIDI score, followed closely by PIDGT-42 and PIDGT-29, indicating their greater similarity to the ideal genotype. Other promising genotypes included PIDGT-35, PIDGT-27, and DT-19. These accessions exhibited favourable combinations of yield and associated traits, demonstrating the usefulness of MGIDI for identifying balanced performers rather than genotypes excelling in only a single trait. Similar effectiveness of MGIDI for simultaneous multi-trait selection has previously been reported in tomato [41]. Comparison of selected genotypes with the overall mean further confirmed the efficiency of MGIDI-based selection. The selected accessions exhibited substantial improvements in fruit weight and yield per plant, indicating the successful identification of superior-yielding genotypes. The selected genotypes may serve as valuable parental resources for future breeding programs aimed at simultaneously enhancing yield, earliness, fruit quality, and adaptability to protected-cultivation conditions [42].

The factor contribution analysis further highlighted differences in the trait architecture of selected genotypes. The genotype PIDGT-39 exhibited balanced performance across most traits, whereas some of the selected genotypes showed specific weaknesses in fruit quality attributes. This information is particularly valuable for designing strategic hybridization programmes aimed at combining complementary traits [43]. For instance, crossing high-yielding genotypes with accessions possessing superior biochemical quality may facilitate the development of hybrids that combine productivity with enhanced nutritional value under protected cultivation systems [42].

Future studies integrating multi-location evaluation, genotype × environment interaction analysis, and genomics-assisted approaches would further strengthen the identification of stable, high-yielding, and nutritionally superior tomato genotypes suitable for protected cultivation systems.

## 5. Conclusions

The comprehensive analysis of pooled data from both years revealed that the evaluated indeterminate tomato inbred lines exhibit substantial genetic variability, particularly in yield, number of fruits per cluster, and fruit weight. The study established that fruit weight and the number of clusters per plant are the most reliable and effective direct selection criteria for improving tomato yield under protected conditions. Based on the results of the present experiment, parental selection strategies should target Cluster VI to combine earliness with high yield, and Cluster V to enhance nutritional quality traits. The monotypic Cluster VII, represented by PIDGT-65, serves as a unique resource for increasing fruit number per plant, locule number per fruit, pericarp thickness, and fruit size. The genotypes identified by the MGIDI, namely PIDGT-39, PIDGT-42, and PIDGT-29, represent elite lines that optimally combine high yield with desirable morphological characteristics. Thus, these genotypes are recommended for commercial cultivation under naturally ventilated polyhouse conditions and as parents in future trait-specific breeding programs to develop high-yielding tomato hybrids that are widely adaptable to adverse climatic conditions.

## Figures and Tables

**Table 1:** Pooled analysis of variance (mean squares) for sixteen morphological, yield, and fruit-quality traits in 57 indeterminate tomato inbred lines evaluated under protected cultivation.

Parameters	Replication	Environment (E)	Genotype (G)	G × E	Error
Degree of freedom (df)	4	1	56	56	224
Plant height (cm)	29.229	1662.548 **	2873.356 **	49.086	128.278
Days to 50% flowering	2.050	19.651 *	132.114 **	4.005	3.058
Number of flowers per cluster	1.642 **	0.920	176.287 **	14.683 **	0.362
Number of clusters per plant	0.772	9.705 **	26.468 **	0.358	0.883
Number of fruits per cluster	1.214	298.482 **	163.578 **	17.607 **	0.688
Number of fruits per plant	22.655	20,799.839 **	30,675.281 **	1736.608 **	114.821
Fruit length (cm)	0.029	0.005	5.629 **	0.011	0.060
Fruit width (cm)	0.001	0.072	11.045 **	0.013	0.046
Pericarp thickness (cm)	0.008	0.024 *	0.332 **	0.002	0.004
Fruit weight (g)	224.050 *	201.465	10,342.574 **	25.164	78.695
Locules per fruit	0.094	0.301	7.359 **	0.059	0.078
Yield per plant (kg)	1.827	78.457 **	84.203 **	9.755 **	1.099
Total soluble solids (oB)	0.885 *	0.561	6.313 **	0.054	0.289
Lycopene content (mg/100 g fresh weight)	1.042	0.001	21.265 **	0.639	0.592
Titratable acidity (%)	0.000	0.000	0.079 **	0.003 **	0.000
Ascorbic acid content (mg/100 g fresh weight)	0.906	10.997 *	330.020 **	4.478 **	2.189

ANOVA was performed using data pooled over two growing seasons (2022–2023 and 2023–2024). E = Environment (growing season); G = Genotype; G × E = Genotype × Environment interaction. Values presented are mean squares from the pooled analysis of variance. *, ** Significant at *p* ≤ 0.05 and *p* ≤ 0.01, respectively.

**Table 2:** Estimates of genetic parameters for different agro-morphological, fruit yield, and quality traits of tomato assessed under protected conditions during 2022–2023 and 2023–2024.

Traits	Mean	Genotypic Coefficient of Variation (%)	Phenotypic Coefficient of Variation (%)	Broad Sense Heritability (%)	Genetic Advance at 5% Selection Intensity	Genetic Advance over Percent of Mean (%)
Plant height (cm)	282.73	7.67	7.85	95.66	43.71	15.46
Days to 50% flowering	45.37	10.19	10.34	96.97	9.37	20.66
Number of flowers per cluster	10.30	50.40	52.64	91.67	10.24	99.40
Number of clusters per plant	8.76	23.80	24.20	96.73	4.23	48.22
Number of fruits per cluster	8.07	61.11	64.69	89.24	9.60	118.91
Number of fruits per plant	77.19	89.97	92.63	94.34	138.96	180.01
Fruit length (cm)	4.82	20.07	20.18	98.95	1.98	41.14
Fruit width (cm)	4.93	27.48	27.54	99.58	2.79	56.49
Pericarp thickness (cm)	0.68	34.44	34.63	98.88	0.48	70.54
Fruit weight (g)	83.21	49.84	50.03	99.24	85.10	102.28
Locules per fruit	3.72	29.68	29.84	98.94	2.26	60.81
Yield per plant (kg)	4.78	73.77	78.45	88.42	6.82	142.89
Total soluble solids (°Brix)	5.50	18.57	19.00	95.58	2.06	37.40
Lycopene content (mg/100 g fresh weight)	9.94	18.65	18.94	96.99	3.76	37.84
Titratable acidity (%)	0.44	25.66	26.13	96.42	0.23	51.91
Ascorbic acid content (mg/100 g fresh weight)	21.26	34.65	34.89	98.64	15.07	70.89

**Table 3:** Genotypic correlation coefficients of yield per plant with various plant morphological, yield, and fruit-quality traits observed in 57 tomato genotypes evaluated under protected conditions over two consecutive years.

Trait	PH	DFF	NFWPC	NCPP	NFPC	NFPP	FL	FW	PT	FWT	LPF	TSS	LYC	TA	AA	YPP
**PH**	1.000 **	−0.540 **	0.189 *	0.155 *	0.242 **	0.227 **	−0.196 *	−0.345 **	−0.290 **	−0.053	−0.292 **	0.232 **	−0.087	0.212 **	0.071	0.084
**DFF**		1.000 **	0.010	−0.289 **	−0.049	−0.082	−0.016	0.293 **	0.068	−0.019	0.227 **	−0.033	0.031	−0.189 *	−0.009	−0.229 **
**NFWPC**			1.000 **	0.507 **	0.926 **	0.907 **	−0.555 **	−0.606 **	−0.605 **	−0.519 **	−0.422 **	0.719 **	0.380 **	0.788 **	0.703 **	0.052
**NCPP**				1.000 **	0.489 **	0.674 **	−0.452 **	−0.515 **	−0.377 **	−0.244 **	−0.347 **	0.365 **	0.293 **	0.488 **	0.297 **	0.340 **
**NFPC**					1.000 **	0.960 **	−0.568 **	−0.565 **	−0.610 **	−0.512 **	−0.416 **	0.746 **	0.372 **	0.773 **	0.746 **	0.079
**NFPP**						1.000 **	−0.613 **	−0.619 **	−0.628 **	−0.514 **	−0.442 **	0.732 **	0.434 **	0.769 **	0.701 **	0.088
**FL**							1.000 **	0.613 **	0.692 **	0.683 **	0.522 **	−0.538 **	−0.276 **	−0.513 **	−0.475 **	0.231 **
**FW**								1.000 **	0.671 **	0.600 **	0.597 **	−0.534 **	−0.282 **	−0.559 **	−0.335 **	0.147
**PT**									1.000 **	0.604 **	0.492 **	−0.583 **	−0.258 **	−0.584 **	−0.529 **	0.234 **
**FWT**										1.000 **	0.412 **	−0.465 **	−0.236 **	−0.441 **	−0.430 **	0.617 **
**LPF**											1.000 **	−0.434 **	−0.164*	−0.421 **	−0.254 **	0.050
**TSS**												1.000 **	0.286 **	0.662 **	0.670 **	−0.075
**LYC**													1.000 **	0.285 **	0.321 **	−0.024
**TA**														1.000 **	0.617 **	0.034
**AA**															1.000 **	−0.002

PH: Plant height (cm); DFF: days to 50% flowering; NFWPC: number of flowers per cluster; NCPP: number of clusters per plant; NFPC: number of fruits per cluster; NFPP: number of fruits per plant; FL: fruit length (cm); FW: fruit width (cm); PT: pericarp thickness (cm); FWT: fruit weight (g); LPF: locules per fruit; TSS: total soluble solids (°Brix); LYC: lycopene content (mg/100 g); TA: titratable acidity (%); AA: ascorbic acid content (mg/100 g); YPP: yield per plant (kg). * Significant at 5% level; ** significant at 1% level.

**Table 4:** Path coefficient analysis between yield, its components, and fruit quality traits in tomato at the genotypic level.

Traits	PH	DFF	NFWPC	NCPP	NFPC	NFPP	FL	FW	PT	FWT	LPF	TSS	LYC	TA	AA	YPP
**PH**	**−0.0769**	0.0415	−0.0145	−0.0119	−0.0186	−0.0175	0.0151	0.0265	0.0223	0.0041	0.0225	−0.0178	0.0067	−0.0163	−0.0055	0.0838
**DFF**	0.0494	**−0.0913**	−0.0009	0.0264	0.0045	0.0075	0.0014	−0.0268	−0.0062	0.0017	−0.0208	0.0031	−0.0028	0.0173	0.0008	−0.2294 **
**NFWPC**	0.0234	0.0012	**0.1238**	0.0628	0.1146	0.1123	−0.0687	0.0751	−0.075	−0.0643	−0.0522	0.089	0.047	0.0976	0.0871	0.0524
**NCPP**	0.1355	−0.2525	0.4436	**0.8745**	0.4274	0.5896	−0.3948	−0.4506	−0.3297	−0.2136	−0.3036	0.3194	0.2564	0.427	0.2595	0.3395 **
**NFPC**	0.4724	−0.096	0.742	0.9559	**0.811**	0.795	−0.713	−0.708	−0.767	−0.699	−0.8129	0.834	0.7282	0.849	0.822	0.0787
**NFPP**	−0.447	0.1621	−0.788	−0.735	−0.806	**−0.851**	0.724	0.759	0.773	0.694	0.8691	−0.818	−0.8527	−0.845	−0.792	0.0879
**FL**	0.021	0.0017	0.0593	0.0482	0.0606	0.0655	**−0.1068**	−0.0655	−0.074	−0.0729	−0.0558	0.0574	0.0294	0.0548	0.0507	0.2307 **
**FW**	−0.0035	0.003	−0.0062	−0.0052	−0.0057	−0.0063	0.0062	**0.0102**	0.0068	0.0061	0.0061	−0.0054	−0.0029	−0.0057	−0.0034	0.1471
**PT**	−0.0186	0.0044	−0.0389	−0.0242	−0.0392	−0.0403	0.0444	0.0431	**0.0642**	0.0388	0.0316	−0.0374	−0.0166	−0.0375	−0.034	0.2338 **
**FWT**	−0.0461	−0.0162	−0.4479	−0.2107	−0.4418	−0.4437	0.5889	0.5178	0.5212	**0.8627**	0.3551	−0.4015	−0.2037	−0.3808	−0.371	0.6166 **
**LPF**	0.018	−0.014	0.026	0.0214	0.0256	0.0272	−0.0322	−0.0368	−0.0303	−0.0253	**−0.0616**	0.0267	0.0101	0.0259	0.0156	0.0503
**TSS**	−0.0179	0.0026	−0.0553	−0.0281	−0.0574	−0.0564	0.0414	0.0411	0.0449	0.0358	0.0334	**−0.077**	−0.022	−0.0509	−0.0516	−0.0751
**LYC**	−0.0019	0.0007	0.0084	0.0065	0.0082	0.0096	−0.0061	−0.0062	−0.0057	−0.0052	−0.0036	0.0063	**0.0221**	0.0063	0.0071	−0.0239
**TA**	−0.0267	0.0238	−0.099	−0.0614	−0.0971	−0.0967	0.0645	0.0702	0.0734	0.0555	0.0528	−0.0831	−0.0358	**−0.1256**	−0.0775	0.0343
**AA**	0.0028	−0.0003	0.0276	0.0116	0.0292	0.0275	−0.0186	−0.0131	−0.0207	−0.0169	−0.0099	0.0262	0.0126	0.0242	**0.0392**	−0.0018

PH: Plant height (cm); DFF: days to 50% flowering; NFWPC: number of flowers per cluster; NCPP: number of clusters per plant; NFPC: number of fruits per cluster; NFPP: number of fruits per plant; FL: fruit length (cm); FW: fruit width (cm); PT: pericarp thickness (cm); FWT: fruit weight (g); LPF: locules per fruit; TSS: total soluble solids (°Brix); LYC: lycopene content (mg/100 g); TA: titratable acidity (%); AA: ascorbic acid content (mg/100 g); YPP: yield per plant (kg). Diagonal values in bold depict direct effects and off-diagonal values depict indirect effects on yield per plant; the last column (YPP) denotes the correlation coefficient with yield per plant at the genotypic level. * Significant at 5% level of significance; ** significant at 1% level of significance; Residual effect: 0.254.

**Table 5:** Contribution of different agro-morphological, yield, and fruit-quality traits to principal components as observed in 57 genotypes of tomato evaluated over two years under protected conditions.

PC	Standard Deviation	Proportion of Variance	Cumulative Proportion
PC1	2.842	0.505	0.505
PC2	1.449	0.131	0.636
PC3	1.235	0.095	0.731
PC4	0.958	0.057	0.789
PC5	0.840	0.044	0.833
PC6	0.756	0.036	0.869
PC7	0.737	0.034	0.903
PC8	0.635	0.025	0.928
PC9	0.576	0.021	0.949
PC10	0.526	0.017	0.966
PC11	0.465	0.013	0.979
PC12	0.370	0.009	0.988
PC13	0.309	0.006	0.994
PC14	0.249	0.004	0.998
PC15	0.180	0.002	1.000

**Table 6:** Contribution of different agro-morphological, yield, and fruit-quality traits to the principal components estimated in 57 indeterminate tomato genotypes.

Traits	PC1	PC2	PC3	PC4	PC5
Plant height (cm)	0.15	6.90	11.65	14.93	0.84
Days to 50% flowering	0.04	11.86	6.76	0.40	32.89
Number of flowers per cluster	1.29	0.03	1.39	0.49	3.66
Number of clusters per plant	0.69	4.66	2.28	14.04	4.05
Number of fruits per cluster	1.32	0.11	0.96	2.03	1.77
Number of fruits per plant	1.35	0.33	1.66	0.01	1.45
Fruit length (cm)	0.95	0.96	1.39	4.89	3.62
Fruit width (cm)	0.94	0.40	5.08	6.71	0.00
Pericarp thickness (cm)	1.02	0.61	1.93	0.02	0.02
Fruit weight (g)	0.74	5.44	4.83	1.64	1.66
Locules per fruit	0.55	0.33	6.16	8.85	9.08
Yield per plant (kg)	0.02	15.10	9.11	1.44	5.12
Total soluble solids (°Brix)	1.20	0.14	0.97	6.48	1.12
Lycopene content (mg/100 g of fresh weight)	0.37	0.18	6.19	19.12	72.79
Titratable acidity (%)	0.94	0.08	1.64	7.62	0.00
Ascorbic acid (mg/100 g of fresh weight)	0.81	0.53	3.51	20.18	3.72

**Table 7:** Cluster means for sixteen agro-morphological, yield and fruit-quality traits of tomato.

Trait	Cluster-I	Cluster-II	Cluster-III	Cluster-IV	Cluster-V	Cluster-VI	Cluster-VII
Plant height (cm)	297.40	296.29	273.61	285.63	264.73	315.96	280.50
Days to 50% flowering	43.50	46.40	47.04	45.78	48.24	34.92	38.34
Number of flowers per cluster	17.33	26.90	7.29	8.20	9.64	8.97	12.67
Number of clusters per plant	11.10	12.28	7.84	8.27	7.70	9.30	16.59
Number of fruits per cluster	15.21	24.15	5.59	6.42	5.68	7.47	9.92
Number of fruits per plant	168.26	295.60	43.31	53.30	43.86	68.98	161.70
Fruit length (cm)	2.82	3.25	5.11	5.25	4.72	5.51	4.68
Fruit width (cm)	2.73	2.70	5.88	5.70	4.71	3.33	4.78
Pericarp thickness (cm)	0.30	0.20	0.71	0.83	0.69	0.72	0.82
Fruit weight (g)	28.28	9.59	93.43	112.07	56.67	93.02	159.42
Locules per fruit	2.17	2.52	4.61	3.77	3.39	3.65	3.08
Yield per plant (kg)	4.05	2.80	3.97	5.94	2.22	6.47	25.80
Total soluble solids (°Brix)	7.45	8.03	5.20	5.06	5.14	5.18	5.05
Lycopene content (mg/100 g fresh weight)	10.25	13.15	9.80	9.41	9.67	9.31	13.07
Titratable acidity (%)	0.63	0.70	0.39	0.41	0.40	0.44	0.42
Ascorbic acid (mg/100 g fresh weight)	31.85	36.99	22.71	15.70	20.37	16.35	20.69

**Table 8:** Contribution of various traits towards genetic divergence observed in 57 tomato genotypes evaluated under protected conditions over two consecutive years.

Sl. No.	Traits	Contribution (%)	Times Ranked 1st
1	Number of flowers per cluster	23.43	374
2	Fruit width (cm)	15.29	244
3	Titratable acidity (%)	13.16	210
4	Locules per fruit	12.34	197
5	Fruit weight (g)	10.40	166
6	Ascorbic acid (mg/100 g fresh weight)	5.20	83
7	Pericarp thickness (cm)	5.14	82
8	Yield per plant (kg)	4.57	73
9	Lycopene content (mg/100 g fresh weight)	3.45	55
10	Days to 50% flowering	3.07	49
11	Number of fruits per cluster	1.32	21
12	Plant height (cm)	0.88	14
13	Fruit length (cm)	0.88	14
14	Number of fruits per plant	0.69	11
15	Number of clusters per plant	0.19	3
16	Total soluble solids (°B)	0.00	0

**Table 9:** Ranking and selection of tomato genotypes based on MGIDI scores based on 16 morphological, yield, and fruit-quality traits.

Genotypes	MGIDI Score	Rank	Selected
PIDGT-39	13.32	1	Yes
PIDGT-42	13.33	2	Yes
PIDGT-29	13.46	3	Yes
PIDGT-35	13.61	4	Yes
PIDGT-27	13.96	5	Yes
DT-19	13.97	6	Yes
PIDGT-37	14.18	7	Yes
DT-7	14.34	8	Yes
PIDGT-63	14.39	9	Yes
PIDGT-25	14.41	10	Yes
PIDGT-45	14.69	11	Yes
PIDGT-34	14.84	12	Yes

## Data Availability

The data presented in this study are available within the article and its Supplementary Materials.

## Supplementary Materials

The following supporting information can be downloaded at: https://www.mdpi.com/article/10.3390/plants15111760/s1. Table S1: Mean performance of 57 tomato genotypes for different agro-morphological, fruit, and yield traits, based on pooled data of 2022–2023 and 2023–2024 growing seasons under naturally ventilated polyhouse conditions; Table S2: Mean performance of 57 tomato genotypes for different fruit quality and biochemical traits, based on pooled data of 2022–2023 and 2023–2024 growing seasons under naturally ventilated polyhouse conditions; Table S3: Comparison of mean performance and percentage change between the original population and selected tomato genotypes for various morphological, yield, and fruit-quality traits; Figure S1: Scree plot showing the variance explained by different principal components in 57 genotypes of tomato evaluated under protected conditions. Figure S2: Cluster diagram illustrating Mahalanobis Euclidean distances between and within clusters, differentiating 57 genotypes of tomato evaluated under protected conditions over two consecutive years.
