# Supplementary material for: Genetic Variability, Trait Association, and Multi-Trait Selection of New Indeterminate Tomato Genotypes Under Protected Cultivation
Source: Plants (Basel). 2026 Jun 5;15(11):1760. doi: 10.3390/plants15111760 (PMC13259263; doi:10.3390/plants15111760)
Supplement: Supplementary file 1 [file plants-15-01760-s001.zip › plants-4314481-supplementary.pdf]

Table S1. Mean performance of 57 tomato genotypes for different agro-morphological, fruit, and yield traits, based on pooled data of 2022-2023 and 2023-2024 growing seasons under naturally ventilated polyhouse conditions.

Genotype	Plant height (cm)	Days to 50% flowering	Number of flowers per cluster	Number of clusters per plant	Number of fruits per cluster	Number of fruits per plant	Fruit length (cm)	Fruit width (cm)	Fruit weight (g)	Yield per plant (kg)
PIDGT-1	283.03	46.75	8.67	7.47	7.92	59.17	6.06	4.32	114.92	6.79
PIDGT-2	265.19	48.00	7.50	6.83	4.08	28.03	6.32	6.92	87.80	2.48
PIDGT-3	259.80	51.17	7.08	6.10	4.25	26.11	5.66	5.90	85.83	2.26
PIDGT-4	247.08	52.17	10.33	7.57	7.08	53.69	5.54	4.82	79.15	4.23
PIDGT-6	250.30	48.08	6.84	8.62	3.75	32.77	5.27	5.05	105.58	3.48
PIDGT-7	258.13	48.67	7.42	8.58	3.75	32.10	5.85	5.22	116.42	3.74
PIDGT-8	260.18	43.92	11.75	8.53	5.75	49.15	4.55	4.48	58.00	2.86
PIDGT-9	264.12	54.58	14.42	7.12	6.75	47.97	4.01	4.78	57.75	2.79
PIDGT-10	282.40	44.92	8.50	6.07	6.67	40.27	4.50	4.88	70.78	2.85
PIDGT-11	272.19	46.84	7.92	5.27	6.75	35.62	5.46	6.72	63.00	2.26
PIDGT-12	254.47	47.66	7.67	5.64	3.50	19.48	4.46	4.68	55.67	0.65
PIDGT-13	232.83	52.67	9.92	7.17	3.50	25.23	4.67	4.94	65.25	0.87
PIDGT-14	266.20	47.75	10.83	9.75	5.75	55.78	6.00	5.64	58.58	2.69
PIDGT-15	285.36	49.75	8.00	8.47	3.58	30.55	4.65	5.88	80.50	1.92
PIDGT-16	273.36	48.08	8.33	8.15	5.33	43.36	4.04	4.56	53.92	1.84
PIDGT-17	285.93	47.92	7.08	7.43	4.42	32.82	4.53	5.44	87.67	2.25
PIDGT-18	275.55	49.50	8.25	8.37	5.58	46.27	4.38	3.55	34.28	1.18
PIDGT-19	293.53	44.67	7.67	6.95	6.50	45.18	5.26	6.13	114.50	5.15
PIDGT-21	288.53	47.50	5.58	8.69	4.33	37.55	4.34	5.32	57.33	1.70
PIDGT-22	253.81	41.00	5.77	8.39	4.92	41.35	4.27	5.56	65.92	2.20
PIDGT-23	244.28	46.75	7.33	10.16	6.33	64.96	5.70	5.65	117.08	7.56
PIDGT-24	233.36	47.67	7.58	11.08	5.58	61.86	5.29	5.85	100.83	6.23
PIDGT-25	290.89	44.92	8.33	8.51	6.17	52.69	4.71	5.67	109.25	5.78
PIDGT-26	284.49	38.50	7.58	8.75	6.67	58.16	5.03	5.92	99.92	5.79
PIDGT-27	298.21	40.17	9.08	9.28	6.92	64.23	5.05	5.53	75.33	4.85
PIDGT-28	267.20	44.42	9.83	8.12	8.33	67.59	5.10	5.86	77.83	5.27
PIDGT-29	281.98	48.33	6.17	8.16	5.00	41.05	5.28	5.87	92.58	3.82
PIDGT-34	287.17	48.50	7.83	7.55	5.17	39.05	5.20	5.58	142.08	5.57
PIDGT-35	309.12	41.58	8.25	8.87	5.33	46.83	5.27	5.08	103.75	4.87
PIDGT-36	269.26	40.67	7.08	8.22	6.17	50.22	4.82	4.60	43.58	2.21
PIDGT-37	307.81	46.34	10.08	8.40	7.83	65.46	4.17	5.12	105.92	6.98
PIDGT-38	273.76	47.50	6.50	8.40	6.67	55.98	5.12	7.92	164.92	9.24
PIDGT-39	280.79	46.67	10.08	9.58	7.75	74.59	5.81	5.79	135.83	10.28
PIDGT-40	304.22	45.42	7.83	6.46	7.33	47.49	4.69	5.06	60.50	2.89
PIDGT-42	295.84	46.25	9.17	8.69	7.58	65.25	4.09	5.61	69.50	4.58
PIDGT-43	293.57	46.33	6.83	7.34	6.58	48.28	4.97	7.40	65.75	3.16
PIDGT-45	268.50	48.42	7.75	6.24	6.42	39.81	5.85	6.28	141.67	5.67
PIDGT-46	292.34	47.50	8.25	6.21	7.00	43.00	5.65	6.75	139.16	6.00
PIDGT-50	290.68	47.92	6.67	8.01	5.92	47.30	5.33	4.78	91.58	4.36
PIDGT-44	275.96	47.83	8.67	7.85	6.67	51.10	5.87	6.54	139.08	7.17
PIDGT-51	285.12	49.25	30.17	11.57	26.42	302.79	4.27	2.48	8.18	2.49
PIDGT-53	289.71	46.84	25.00	13.62	23.50	318.64	2.45	2.06	10.70	3.42
PIDGT-54	301.86	48.08	27.42	13.47	24.75	331.75	2.59	2.14	7.92	2.64
PIDGT-55	303.64	39.92	17.58	11.19	14.67	163.30	2.36	2.43	10.51	1.71

PIDGT-57	308.47	41.42	25.00	10.48	21.92	229.23	3.70	4.11	11.58	2.65
PIDGT-60	295.75	39.42	16.67	13.24	15.33	202.44	2.53	2.53	9.55	1.93
PIDGT-61	289.70	46.17	17.50	9.21	14.33	132.39	4.15	3.87	84.89	11.08
PIDGT-62	304.15	48.00	7.25	8.93	6.33	55.59	4.65	4.88	153.75	8.60
PIDGT-63	300.50	48.50	17.58	10.75	16.50	174.92	2.25	2.10	8.18	1.47
PIDGT-65	280.50	38.34	12.67	16.59	9.92	161.70	4.68	4.78	159.42	25.80
PIDGT-66	303.45	45.67	7.83	6.71	6.67	44.48	5.91	5.79	162.17	7.30
PIDGT-92	271.71	46.42	7.00	10.31	6.83	70.42	4.87	5.78	61.77	4.32
DT-1	309.36	36.08	8.33	10.35	6.92	71.01	4.74	2.78	92.34	6.59
DT-4	317.48	33.42	7.58	8.15	7.00	56.84	5.55	3.67	92.08	5.26
DT-7	347.01	34.33	8.42	8.79	6.83	58.88	5.85	3.22	98.58	5.82
DT-9	292.58	34.50	11.50	10.01	8.75	86.34	5.86	3.15	103.75	9.03
DT-19	313.37	36.25	9.00	9.18	7.83	71.85	5.54	3.83	78.34	5.62
Overall Mean	282.73	45.37	10.30	8.76	8.07	77.19	4.82	4.93	83.21	4.78
C.D. @ 5%	12.89	1.99	0.68	1.07	0.94	12.19	0.28	0.24	10.09	1.19

Table S2. Mean performance of 57 tomato genotypes for different fruit quality and biochemical traits, based on pooled data of 2022-2023 and 2023-2024 growing seasons under naturally ventilated polyhouse conditions.

Genotype	Pericarp thickness (cm)	Locules per fruit	Total soluble solids (oB)	Lycopene content (mg/100 g)	Titratable acidity (%)	Ascorbic acid (mg/100 g)
PIDGT-1	0.48	4.67	5.26	8.46	0.33	25.99
PIDGT-2	1.12	5.17	5.08	9.02	0.47	19.88
PIDGT-3	0.51	5.00	5.03	9.94	0.47	15.74
PIDGT-4	0.82	4.58	5.28	8.62	0.41	17.61
PIDGT-6	0.78	4.58	4.99	9.25	0.46	19.68
PIDGT-7	0.63	5.75	5.37	11.41	0.30	18.15
PIDGT-8	0.67	4.75	4.98	12.09	0.39	23.63
PIDGT-9	0.72	4.42	4.92	8.15	0.34	17.61
PIDGT-10	0.90	4.42	5.49	13.12	0.38	21.73
PIDGT-11	0.91	4.42	5.39	11.07	0.26	21.96
PIDGT-12	0.54	2.25	5.04	13.14	0.39	18.05
PIDGT-13	0.66	2.92	5.69	9.10	0.39	19.95
PIDGT-14	1.06	4.75	4.56	9.11	0.36	17.20
PIDGT-15	0.62	4.75	5.05	9.99	0.45	18.56
PIDGT-16	0.66	2.92	5.39	7.29	0.49	21.64
PIDGT-17	0.61	4.25	4.58	8.54	0.34	21.79
PIDGT-18	0.46	2.17	5.36	12.32	0.37	16.59
PIDGT-19	0.68	4.25	5.05	9.93	0.31	22.14
PIDGT-21	0.58	4.08	5.48	10.23	0.32	23.79
PIDGT-22	0.74	4.08	5.28	11.34	0.45	22.71
PIDGT-23	1.00	4.75	5.08	12.13	0.42	18.02
PIDGT-24	0.80	3.08	4.71	8.11	0.41	22.20
PIDGT-25	0.76	6.08	5.18	8.03	0.44	14.41
PIDGT-26	0.77	2.33	5.45	8.39	0.38	17.77
PIDGT-27	0.83	2.25	4.87	8.13	0.39	13.96
PIDGT-28	0.62	3.83	4.63	10.82	0.39	17.55
PIDGT-29	0.92	3.92	4.83	11.85	0.38	13.46
PIDGT-34	0.82	3.25	4.86	11.37	0.50	14.84
PIDGT-35	0.39	2.42	4.77	8.04	0.46	13.61
PIDGT-36	0.58	2.92	4.88	8.21	0.49	25.48
PIDGT-37	0.56	4.08	4.57	9.91	0.40	14.18
PIDGT-38	0.77	5.58	4.84	9.86	0.54	25.83
PIDGT-39	0.83	4.92	4.58	7.18	0.43	13.32
PIDGT-40	0.74	2.25	5.31	8.64	0.35	25.93
PIDGT-42	0.88	3.42	4.82	8.32	0.34	13.34
PIDGT-43	0.82	6.25	4.89	8.70	0.37	26.78
PIDGT-45	1.07	4.25	5.32	7.59	0.38	14.69
PIDGT-46	0.74	4.33	6.64	8.70	0.36	34.25
PIDGT-50	0.83	3.25	5.85	10.62	0.45	14.90
PIDGT-44	0.90	3.84	5.50	11.81	0.38	15.60
PIDGT-51	0.14	2.50	7.87	12.21	0.77	38.68
PIDGT-53	0.18	2.25	8.13	14.03	0.64	34.75
PIDGT-54	0.16	2.08	8.32	14.98	0.60	38.30
PIDGT-55	0.22	2.25	7.75	9.11	0.62	37.44
PIDGT-57	0.31	3.25	7.81	11.39	0.77	36.25
PIDGT-60	0.22	2.25	8.37	13.30	0.73	34.01
PIDGT-61	0.51	2.08	8.03	10.38	0.75	41.59
PIDGT-62	0.97	3.25	5.64	8.68	0.31	16.04
PIDGT-63	0.26	2.08	5.67	8.20	0.43	14.39
PIDGT-65	0.82	3.08	5.05	13.07	0.42	20.69
PIDGT-66	0.83	3.08	5.00	8.08	0.44	21.29
PIDGT-92	0.84	4.25	5.10	8.18	0.48	22.03
DT-1	0.75	3.33	5.06	9.27	0.47	14.98
DT-4	0.61	4.25	4.99	10.22	0.43	20.21
DT-7	0.72	3.08	5.31	7.66	0.37	14.35
DT-9	0.92	4.25	5.35	9.90	0.48	18.25
DT-19	0.58	3.33	5.18	9.52	0.45	13.97
Overall Mean	0.68	3.72	5.50	9.94	0.44	21.26

S.E. (m) ±	0.02	0.11	0.22	0.31	0.01	0.60
C.D. @ 5%	0.07	0.32	0.61	0.88	0.02	1.68

Table S3. Comparison of mean performance and percentage change between the original population and selected tomato genotypes for various morphological, yield, and fruit-quality traits.

Trait	Original Population	Selected Genotypes	% Change
Plant height (cm)	282.73	298.43	5.55
Days to 50% flowering	45.37	44.19	-2.60
Number of flowers per cluster	10.30	9.31	-9.57
Number of clusters per plant	8.76	8.67	-1.11
Number of fruits per cluster	8.07	7.44	-7.78
Number of fruits per plant	77.19	66.22	-14.22
Fruit length (cm)	4.82	4.92	2.12
Fruit width (cm)	4.93	4.97	0.79
Pericarp thickness (cm)	0.68	0.72	5.47
Fruit weight (g)	83.21	96.75	16.28
Locules per fruit	3.72	3.59	-3.39
Yield per plant (kg)	4.78	5.44	14.00
Total soluble solids (oB)	5.50	5.00	-9.15
Lycopene content (mg/100 g fresh weight)	9.94	8.82	-11.33
Titratable acidity (%)	0.44	0.41	-5.95
Ascorbic acid (mg/100 g fresh weight)	21.26	14.04	-33.95

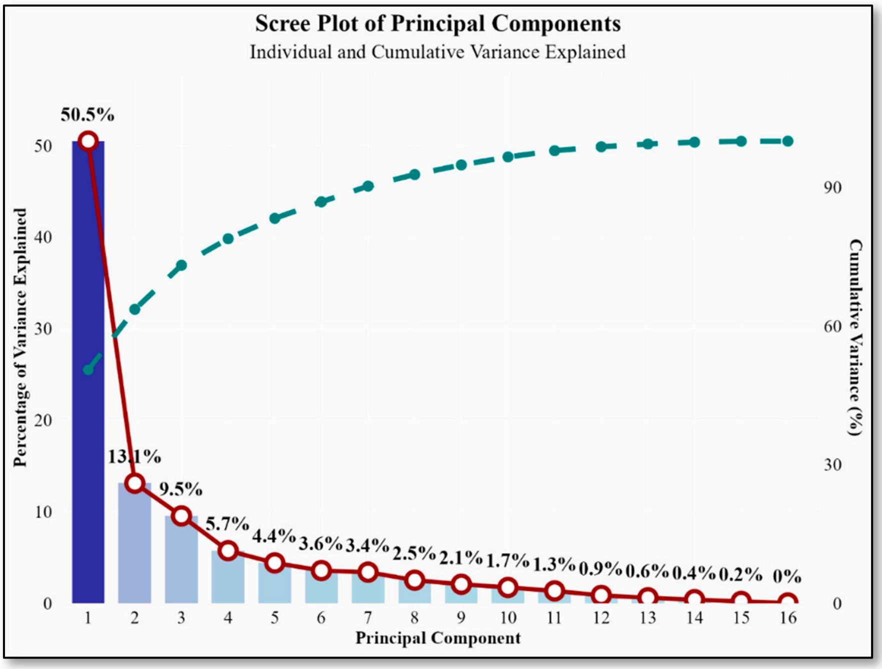

Figure S1. Scree plot showing the variance explained by different principal components in 57 genotypes of tomato evaluated under protected conditions.

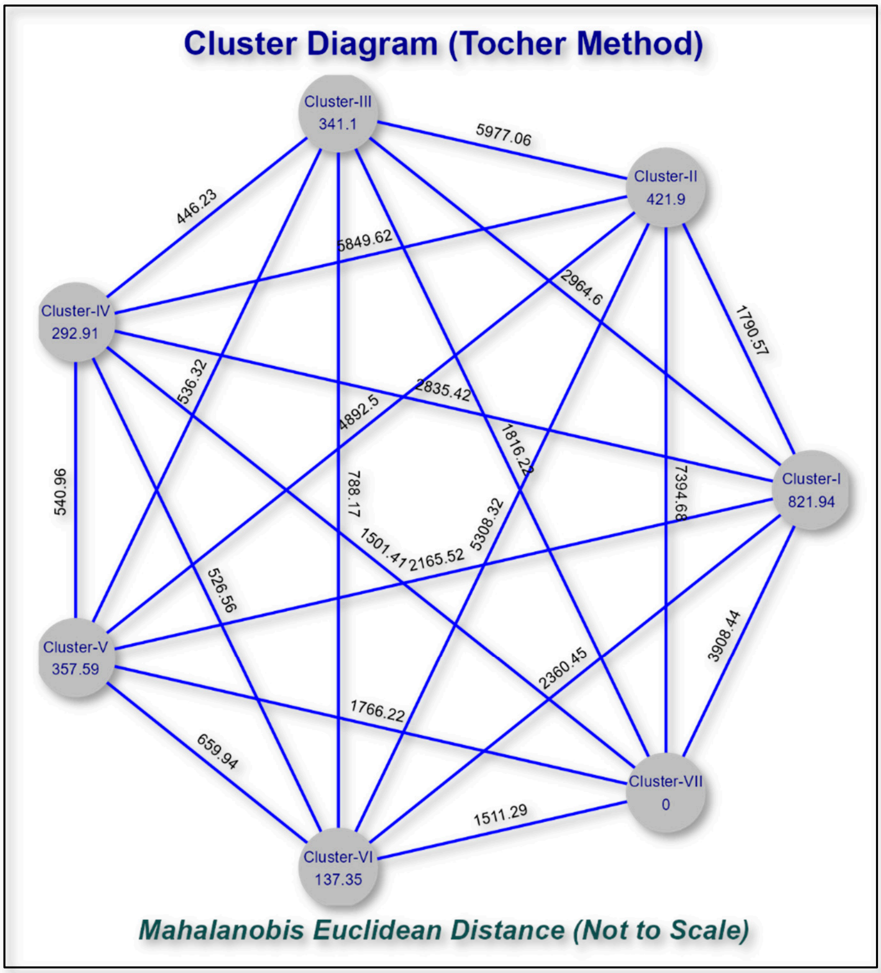

Figure S2. Cluster diagram illustrating Mahalanobis Euclidean distances between and within clusters, differentiating 57 genotypes of tomato evaluated under protected conditions over two consecutive years.
